# HLA Polymorphisms Linked to the Severity and Extent of Periodontitis in Patients with Type 1 Diabetes from a Brazilian Mixed Population

**DOI:** 10.3390/ijerph22040512

**Published:** 2025-03-27

**Authors:** Carlos Felipe Sousa Menezes, Lucas Meneses Lage, Luís Gustavo Souza Santos, Gilvan Cortês Nascimento, Marcelo Magalhães, Alexandre Facundo, Dayse Aparecida Silva, Luís Cristóvão Porto, Marília Brito Gomes, Manuel dos Santos Faria, Rossana Sousa Azulay, Vandilson Rodrigues

**Affiliations:** 1 School of Dentistry, Federal University of Maranhão, São Luís 65085-580, MA, Brazil; carlos.fsm@discente.ufma.br (C.F.S.M.); lucas.lage@discente.ufma.br (L.M.L.); souza.luis@discente.ufma.br (L.G.S.S.); 2 Research Group in Clinical Endocrinology and Molecular Metabolism, São Luís 65020-070, MA, Brazil; gilvan.cn@ufma.br (G.C.N.); magalhaes_ms@yahoo.com.br (M.M.); alexandrenfacundo@hotmail.com (A.F.); mfaria1949@gmail.com (M.d.S.F.); rossana.azulay@ufma.br (R.S.A.); 3 School of Medicine, Federal University of Maranhão, São Luís 65020-240, MA, Brazil; 4 Graduate Program in Adult Health, Federal University of Maranhão, São Luís 65085-580, MA, Brazil; 5 DNA Diagnostic Laboratory, Rio de Janeiro State University, Rio de Janeiro 20550-900, RJ, Brazil; dayse.a.silva@gmail.com (D.A.S.); luis.cristovaoporto@gmail.com (L.C.P.); 6 Diabetes Unit, State University of Rio de Janeiro, Rio de Janeiro 20551-030, RJ, Brazil; mariliabgomes@gmail.com

**Keywords:** human leukocyte antigen, type 1 diabetes, periodontal diseases

## Abstract

This study aimed to investigate the relationship between Class II human leukocyte antigen (HLA) alleles (DRB1, DQA1, and DQB1) and the severity and extent of periodontitis in patients with Type 1 diabetes (T1D). A cross-sectional study was conducted with 49 patients with T1D. Demographic data and diabetes history were collected. A clinical examination was performed to assess periodontal variables. The patients were categorized by the periodontitis severity and the extent of periodontitis. Peripheral blood samples were analyzed to identify the percentage of autosomal ancestry (Native American, European, and African) and the HLA-DRB1*, HLA-DQA1*, and HLA-DQB1* alleles. The DRB1*03 and DRB1*15 haplogroups were significantly associated with an increased risk of generalized periodontitis (OR = 19.8, 95% CI = 1.14–346, *p* = 0.003; OR = 41.2, 95% CI = 1.85–917, *p* < 0.001) and severe periodontitis (OR = 7.7, 95% CI = 1.68–35.5, *p* = 0.003; OR = 21.2, 95% CI = 0.97–461, *p* = 0.005). No associations were observed between the HLA-DQA1 and HLA-DQB1 alleles and periodontitis. These findings suggest that patients with T1D from a highly mixed Brazilian population carrying the DRB1*03 and DRB1*15 haplogroups are at higher risk for developing more severe and generalized forms of periodontitis.

## 1. Introduction

Periodontitis is a common disease that negatively affects oral health and quality of life [1,2,3]. It is characterized by chronic inflammation that can lead to destruction of the supporting dental tissues [4]. In addition to its local effects, periodontal inflammation is a well-established risk factor for poor glycemic control in patients with diabetes, further complicating their health management [5]. Type 1 diabetes (T1D), which results from autoimmune destruction of the pancreatic β-cells responsible for insulin production, is a condition that has a complex relationship with periodontal disease [6]. Although the exact mechanisms linking diabetes and periodontitis remain unclear, evidence suggests that immune system factors play a central role in this interaction [5].

Genetic factors, particularly those involving the human leukocyte antigen (HLA) system, are known to influence susceptibility to both T1D and periodontitis [7,8,9]. The HLA genes, especially those in the class II region such as HLA-DR and HLA-DQ, are well-established risk factors for autoimmune diseases such as T1D [8,9,10] and have also been implicated in periodontal disease susceptibility [9]. The HLA-DRB1 gene, located within the DR locus, plays a critical role in antigen presentation and activation of the immune system [11]. It interacts with the HLA-DRA gene to form a heterodimer that presents foreign peptides to the immune system, triggering immune responses such as T-cell activation [12]. This pathway is central not only to autoimmune diseases, but also to the body’s response to infection, including periodontal pathogens. Thus, variations in HLA-DRB1 and other class II alleles may contribute to a dysregulated immune response, potentially leading to more severe forms of periodontitis.

The potential role of HLA in periodontal disease is further supported by the fact that gingival fibroblasts, key cells in periodontal tissue, express HLA molecules. These cells can release pro-inflammatory cytokines, including interleukin-6 and MCP-1, which promote inflammation and may contribute to periodontal tissue damage [13]. Thus, HLA may modulate the immune response without directly presenting antigens by signaling that leads to cytokine release [14].

In addition to immune function, genetic profiles, such as variations in the HLA, influence the host response to oral microbiota, a key factor in the development of periodontitis [15]. The interaction between genetic, environmental and bacterial factors contributes to disease progression [16,17,18]. Related to immune responses, HLA has been investigated in studies on periodontal diseases, inflammation, and ancestry diversity in human populations [17,19].

Despite evidence linking HLA alleles to periodontitis [20,21,22], few studies in Brazil have examined this connection, particularly in patients with T1D [23]. The HLA allele polymorphisms vary across populations [24], influencing susceptibility or protection against diseases [7,8]. With its significant ethnic diversity, Brazil has yet to explore the genetic basis of T1D fully [25], which is highly prevalent in adults ages 20–79 years [26]. Understanding genetic risk factors in Brazilian patients with T1D can help improve early intervention and reduce the effect on their quality of life. Untreated periodontal diseases can lead to tooth loss and systemic inflammation, further compromising their quality of life [27]. Early periodontitis diagnosis and treatment are essential, especially for younger patients [28].

This study aims to fill this gap by investigating the association of class II HLA alleles (HLA-DRB1, HLA-DQA1, and HLA-DQB1) with the severity and extent of periodontitis in Brazilian patients with T1D. We hypothesize that specific HLA alleles may be associated with severe forms of periodontitis, potentially highlighting genetic markers that could be used for early detection and personalized management of periodontal disease.

## 2. Materials and Methods

### 2.1. Study Design

This cross-sectional study was conducted with patients diagnosed with T1D under medical monitoring in the University Hospital of the Federal University of Maranhão (HUUFMA), São Luís, Brazil. The study was submitted and approved by the Research Ethics Committee of the HUUFMA (CAAE: 67647823.5.0000.5086). All participants were informed of the study procedures and signed the informed consent form after reading and fully understanding the research.

### 2.2. Sample

The study sample comprised 49 patients screened at the Endocrinology Unit at HUUFMA. Patients over 18 years old of both sexes with T1D who previously participated in an HLA genotyping study and were under clinical follow-up at the hospital were included. Patients were excluded if they were hospitalized during the screening period, had fewer than 10 teeth (to avoid confounding by tooth loss due to causes other than periodontitis, such as dental caries or trauma), were undergoing orthodontic treatment with fixed appliances, had received periodontal treatment in the six months prior to the sample collection, were current or former smokers (within the last six months), or were pregnant or breastfeeding.

### 2.3. Demographic and General Health Data Collection

Demographic data (sex and age) and general health history were collected via a semi-structured questionnaire. Some health history data were retrieved from the hospital records (age at diabetes diagnosis, disease duration, and serum glycosylated hemoglobin or HbA1c level). Serum data were taken as the maximum period of serum collection at the time of the periodontal examination of 7 days.

### 2.4. HLA Allele Determination

A DNA sample was extracted from participants via peripheral blood samples according to the manufacturer’s instructions using the SP QIA Symphony commercial kit (Qiagen, Redwood City, CA, USA). Class II HLA alleles (HLA-DRB1*, HLA-DQA1*, and HLA-DQB1*) were genotyped using PCR-RSSO (high-resolution LABType, One Lambda Inc., West Hills, CA, USA) combined with Luminex technology [29]. The allele definition was based on the common and well-documented (CWD, v3.0) list, and ambiguities were resolved using sequencing methods [30]. Three haplotype frequencies for the loci (DRB1, DQA, and DQB1) were estimated by resolving the phase and allele ambiguity using the expectation-maximization algorithm.

### 2.5. Autosomal Ancestry Percentage

The composition of autosomal ancestry percentage was inferred using a 46 AIM-INDEL panel according to an established protocol [31]. These markers display variable allele frequencies among European, African, and Native American populations. Genotyping was performed using multiplex PCR followed by capillary electrophoresis with the ABI 3500 system. Alleles were named using Gene Mapper software (v.4.1, Applied Biosystems by Life Technologies, Carlsbad, CA, USA). Ancestry was estimated using Structure software (v.2.3.3), with the HGDP-CEPH (H952 subset) reference population panel.

### 2.6. Periodontal Clinical Examination

**Figure 1:**
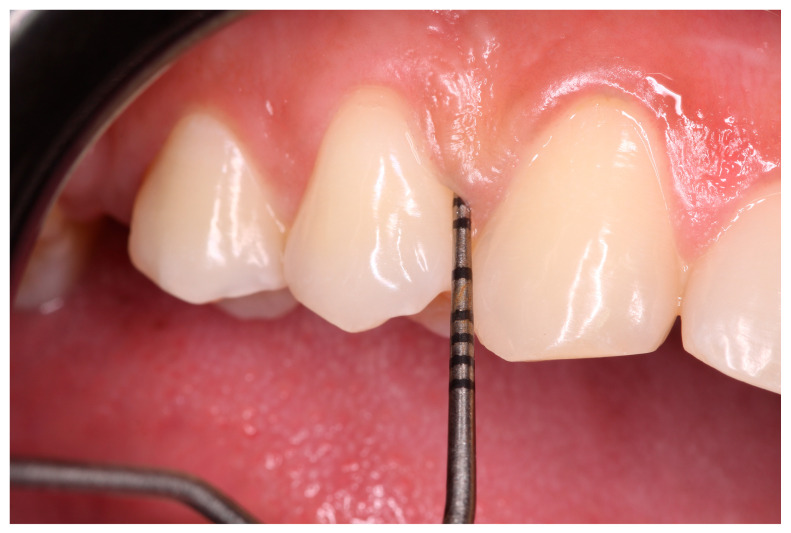
The image illustrates the periodontal probing process in the mesiovestibular region of the upper right first premolar.

A single trained examiner (CFSM) conducted the periodontal examination using sterilized instruments, a clinical mirror, and a HuFriedy-PCPUNC 15 mm probe (HuFriedy^®^, Mfg. Co., Inc., Chicago, IL, USA). The probe was applied with light pressure, as parallel as possible to the long axis of the tooth, with a slight inclination in the interproximal region (Figure 1), recording the probing depth values for all present teeth in each of the following regions: distovestibular, centrovestibular, mesiovestibular, distolingual, centrolingual, and mesiolingual. A prior training session ensured standardized measurement methods for the probing depth and clinical attachment level. The intra-examiner agreement coefficient was 0.85 for the probing depth and 0.82 for the clinical attachment level, indicating excellent agreement.

The following periodontal variables were collected for this study: probing depth, clinical attachment level, gingival bleeding index, and visible plaque index [32]. Periodontal disease severity was classified according to the 2017 World Workshop on the Classification of Periodontal and Peri-Implant Diseases and Conditions, assigning Stages I to IV as follows: Stage I (initial periodontitis), Stage II (moderate periodontitis), Stage III (severe periodontitis with potential for further tooth loss), and Stage IV (advanced periodontitis with extensive tooth loss and potential for complete tooth loss). Periodontitis extent was categorized as localized (<30% of affected teeth) or generalized (≥30% of affected teeth) [33].

### 2.7. Statistical Analysis

The data were analyzed using R software (v.4.4.2; R-Core Team, 2022) and GraphPad Prism version 10.0 (GraphPad Software Inc., San Diego, CA, USA). The descriptive statistics include absolute frequency, percentage, mean, and standard deviation. The percentage of autosomal ancestry was used primarily as a descriptive variable to highlight the genetic diversity within the Brazilian population. The distribution of HbA1c and fasting glucose was assessed using the Shapiro–Wilk test. The Mann–Whitney test was then selected for comparative analysis of serum data. The chi-square (χ^2^) and Fisher’s exact tests were employed to compare frequencies. The odds ratios (OR) and corresponding 95% confidence intervals (CIs) were calculated to estimate the association between Class II HLA alleles and periodontitis outcomes. When zeros caused problems in the OR calculation, 0.5 was added to all cells [34]. All tests applied a significance level of 5%. The power analysis was performed using G*Power software (v.3.1.9.6, University of Kiel, Düsseldorf, Germany). The calculation was based on the following parameters: χ^2^ test, effect size w = 0.06, α = 0.05, sample size = 49, and results for the association measures of DRB103 and DRB1*15 with the study outcomes. The sample demonstrated a power of at least 0.84 for these two markers.

## 3. Results

This study included 49 patients with T1D (23 men and 26 women), with a mean age of 39.7 ± 9.9 years. Regarding self-reported skin color, 71.4% of the sample identified as mixed-race. The mean age at initial diagnosis of T1D was 16.5 ± 10 years. The mean glycemic markers in the sample were 8.0 ± 1.6 for HbA1c and 167 ± 103 for blood glucose. For the periodontal diagnosis, 46.9% of participants had Stage II periodontitis, Stage III was identified in 20.4%, and generalized periodontitis in 12.2%. The mean values for gingival bleeding and visible plaque indices were 4.7 ± 8.1 and 7.4 ± 8.8, respectively (Table 1).

**Figure 2:**
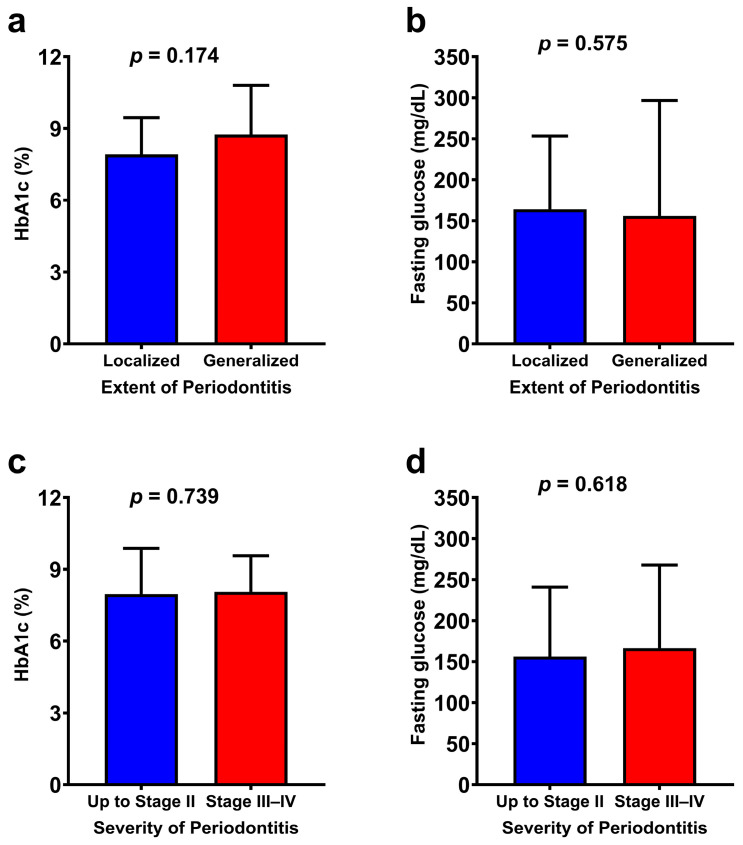
Comparative analysis of HbA1c and fasting glucose levels between the extent (**a**,**b**) and severity (**c**,**d**) categories of periodontitis Continuous data were compared between groups using the Mann–Whitney test.

Additionally, the serum levels of HbA1c and fasting glucose were compared across the categories of extent and severity of periodontitis (Figure 2). No statistically significant differences were found in the serum marker levels between the categories of extent and severity of periodontitis.

Table 2 presents the distribution of the evaluated genetic markers. The composition of autosomal ancestry was highest for the European component (44.6 ± 15.4%), followed by the African component (31.2 ± 13.5%). The three most frequent HLA-DRB1* haplogroups were DRB1*03 (37.8%), DRB1*04 (22.4%), and DRB1*07 (11.2%). The three most frequent HLA-DQA1* alleles were 05:01 (36.5%), 03:01 (26%), and 02:01 (11.5%). The most frequent alleles for HLA-DQB1* were 02:01 (33.7%), 03:01 (24.5%), and 02:02 (15.3%).

Table 3 presents the association analysis of the HLA-DRB1* haplogroups with periodontal outcomes. Patients with the DRB1*03 (OR = 19.8; 95% CI [1.14, 346], *p* = 0.003) and DRB1*15 (OR = 41.2; 95% CI [1.85, 917], *p* < 0.001) haplogroups were associated with a higher risk for generalized periodontitis. These alleles were also associated with severe periodontitis (Stages III–IV): DRB1*03 (OR = 7.71; 95% CI [1.68, 35.5], *p* = 0.003) and DRB1*15 (OR = 21.2; 95% CI [0.97, 461], *p* = 0.005).

**Figure 3:**
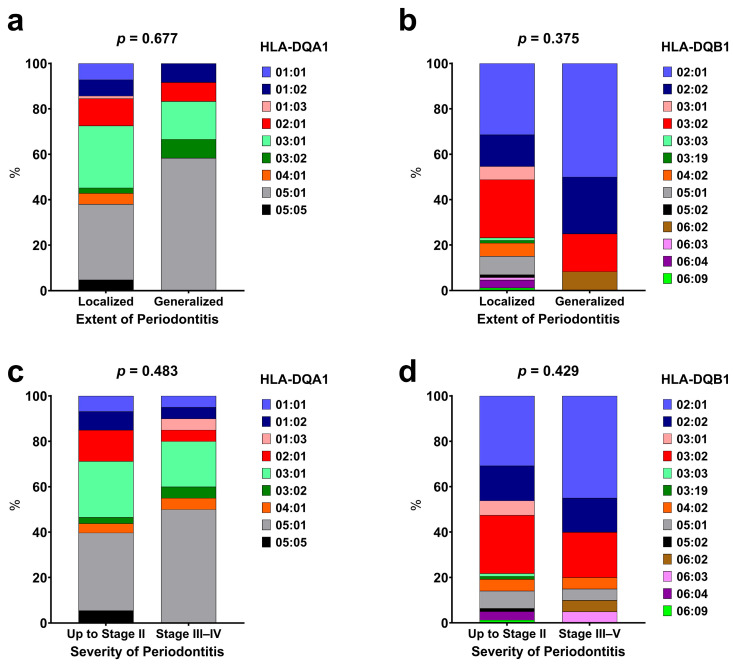
Distribution of HLA-DQA1 and HLA-DQB1 alleles based on the extent (**a**,**b**) and severity (**c**,**d**) of periodontitis. Fisher’s exact test was used to compare categorical data.

No significant associations were observed between the HLA-DQA1* and HLA-DQB1* alleles with the extent (Figure 3a,b) and severity of periodontitis (Figure 3c,d).

## 4. Discussion

Allelic association with a disease occurs when a specific allele is frequently or sporadically significantly present [16]. The primary findings of this study suggest that patients with T1D from a mixed-race Brazilian population who possess the HLA-DRB1*03 or HLA-DRB1*15 haplogroups have a higher risk of developing severe and generalized periodontitis, confirming the hypothesis of this research.

Periodontal diseases are typically associated with the DQ locus [9], and in a study involving Brazilians, the HLA-DRB1* locus was linked to resistance to periodontitis [23]. The current study observed an association between the DRB1 locus and severe and generalized forms of periodontitis in Brazilian patients with T1D, suggesting that this locus may influence the susceptibility to periodontal disease, particularly in individuals with this autoimmune disease.

However, unlike other loci, such as HLA-DQA1* and HLA-DQB1*, which did not display significant associations with the severity or extent of periodontitis in the sample, this work identified an interesting pattern connected to two alleles of the HLA-DRB1 locus: HLA-DRB1*03 and HLA-DRB1*15. This combination of DR locus alleles may imply a complex interaction between genetic factors and the comorbidity of diabetes, modulating the immune response and predisposition to more aggressive forms of periodontitis. This finding underlines the need for more personalized methods in managing periodontal diseases, considering the interaction of genetic factors with systemic conditions, especially in a mixed-race population, such as the Brazilian population, where different genetic variants may act distinctly.

The HLA-DRB1*03 haplogroup has been linked to periodontitis in a population from Iraq without a history of diabetes and was considered a risk factor for periodontitis, with a five-fold increased risk compared to individuals in the same population who did not carry this allele [17]. These data suggest that the HLA-DRB1*03 haplogroup may influence predisposition to periodontitis regardless of comorbidities, such as diabetes, making it an applicable genetic marker in the context of oral health for this population. Furthermore, evidence has shown that the HLA-DRB1*03 allele is associated with an increased risk of T1D in Brazilian samples [29,35]. This finding further supports the hypothesis that the association between T1D and periodontitis may be mediated, at least in part, by genetic factors.

The HLA-DRB1*15:01 allele has been linked to early-onset periodontitis, with the hypothesis that patients with this allele may have an accelerated T-cell response to periodontal pathogens, such as *Porphyromonas gingivalis*, triggering hyperimmune reactions [36]. Moreover, one study found that the HLA-DRB1*15 haplogroup was associated with aggressive generalized periodontitis [37].

The HLA-DRB1*03 haplogroup has been associated with immunogenetic susceptibility to T1D [25], an autoimmune disease with a bidirectional relationship to periodontitis that is considered a risk factor [5]. However, although the HLA-DRB1*15 haplogroup is not directly associated with diabetes as a risk allele but as a protective allele [38], this study found it relevant in patients with diabetes and with severe and generalized periodontitis. This association raises the hypothesis that the severity of periodontitis in these individuals may be influenced by a genetic predisposition conferred by the HLA-DRB1*15 haplogroup, independent of their diabetes status. In contrast, the HLA-DRB1*03 haplogroup correlates significantly with severe and generalized periodontitis in the study sample. This association can be considered an interaction between genetic factors, such as ancestry and autoimmune diseases (e.g., diabetes), which are prevalent in the Brazilian population.

It is important to emphasize that the potential biological effects of the HLA-DRB1*03 and HLA-DRB1*15 polymorphisms in the pathogenesis of periodontitis may be primarily due to their role in the regulation of immune responses. HLA-DRB1 molecules are essential for antigen presentation, particularly the presentation of peptide fragments to T cells, which in turn triggers adaptive immune responses [39,40]. Both HLA-DRB1*03 and HLA-DRB1*15 may modulate these processes by influencing the immune system’s recognition of microbial antigens, ultimately contributing to an exaggerated inflammatory response. In addition, these polymorphisms may interact with genetic and environmental factors, such as an individual’s specific oral microbiome and overall immune health [39]. This interaction may enhance or dysregulate immune responses, thereby promoting a chronic inflammatory state in periodontal tissues. Future studies should focus on understanding how carriers of these specific HLA-DRB1 alleles may exhibit increased production of pro-inflammatory cytokines, including TNF-α, IL-1β, and IL-6, all of which are central to the tissue destruction seen in periodontitis. Investigating these pathways could offer valuable insights into the molecular mechanisms linking genetic susceptibility with periodontal disease progression.

The findings on the HLA-DRB1*03 and HLA-DRB1*15 haplogroups in the study patients are relevant when considering the relationship between severe and generalized periodontitis, systemic diseases (e.g., diabetes), and the ancestry of the individuals. The Brazilian population, characterized by its genetic diversity due to the admixture of ethnic groups, provides a unique basis for studying the genetic determinants of diseases, including periodontitis. The Brazilian sample is highly heterogeneous, resulting from the admixture of European, African, and Native American ethnicities, with this racial mix occurring differently in each region of Brazil [41,42].

In this context, investigating HLA alleles in the study sample collected in the northeast of the country, with highly mixed European, African, and Native American components, may help identify predisposition factors in highly mixed populations with these three ancestral roots, as observed in other Latin American countries. The HLAs are crucial in establishing susceptibility or resistance to periodontal diseases, which varies according to population [22]. This variation may justify the differences in the inflammatory response to microbial challenges [24].

Evidence suggests that susceptibility and resistance to chronic and aggressive periodontitis may be influenced by specific combinations of HLA markers and other genetic factors [17]. The study results indicate that the HLA-DRB1*03 and HLA-DRB1*15 haplogroups may predispose Brazilian patients with T1D to advanced forms of periodontal disease. Thus, these alleles may indicate an increased susceptibility to severe periodontitis in this population, implying the need for more specific and personalized prevention and treatment strategies for individuals with this genetic and clinical profile.

Knowing the genetic profile of patients could facilitate diagnosis, prognosis, and interventions targeting environmental factors that activate genes involved in susceptibility [16]. In addition, identifying these factors may help customize therapy and periodontal monitoring to the needs of each patient. Therefore, such studies are essential to a better understanding of the mechanisms underlying severe periodontitis.

A detailed analysis of HLA in certain populations could clarify whether the severity of periodontitis is predominantly a consequence of diabetes, with its immunological and metabolic basis, or more directly related to the presence of specific HLA alleles that could predispose individuals to an exaggerated inflammatory response. Understanding this interaction between genetics and clinical conditions is vital to defining the critical factors in the manifestation of severe periodontitis and developing strategies for early diagnosis and personalized treatment of patients with this immunogenetic profile.

This study presents an innovative approach to exploring the association between HLA genes, autosomal ancestry, and periodontitis in patients with T1D in Brazil. This pioneering approach offers insight into the genetic influence on susceptibility to periodontitis in a diverse population. Nevertheless, the limitations in the cross-sectional study design and sample size should be considered when interpreting the results.

Although the cross-sectional design of the present study limits the ability to draw conclusions about causality, the genetic exposure (i.e., the presence of specific HLA alleles) precedes the development of periodontal disease, providing valuable insight into the potential role of these genetic factors in the onset of periodontitis. Since HLA alleles are inherited and present from birth, they could influence the immune system’s response to microbial or environmental challenges throughout an individual’s lifetime. In this sense, the presence of certain HLA-DRB1 haplogroups, such as DRB1*03 and DRB1*15, may predispose individuals to an exaggerated inflammatory response when exposed to periodontal pathogens. This finding underscores the importance of understanding genetic risk factors early in life, possibly even before the onset of periodontal disease, to better predict disease progression and develop targeted interventions.

However, due to the cross-sectional nature of the study, we cannot definitively conclude that HLA alleles are directly responsible for the onset of periodontitis. A longitudinal approach that follows patients over time would be crucial for establishing a temporal relationship between genetic predisposition and the development of periodontal disease. By assessing patients at multiple points in their lives, it would be possible to track the onset and progression of periodontitis in relation to the presence of specific HLA alleles, as well as to examine how other factors such as glycemic control, environmental exposures, and oral hygiene practices interact with genetic predisposition over time.

Another limitation of this study is the relatively small sample size, which may have led to wider confidence intervals for the observed associations. While the findings provide valuable insights into the potential genetic factors underlying periodontitis in T1D patients, a larger sample size would improve statistical power and the robustness of these results. Additionally, longitudinal studies could help elucidate the mechanisms underlying the immune response to periodontal pathogens in genetically predisposed individuals, providing more robust evidence for the development of personalized prevention and treatment strategies. Therefore, future research should incorporate longitudinal designs with larger sample sizes and a broader range of genetic profiles to further validate and extend the findings of this study.

## 5. Conclusions

The study findings suggest that patients with T1D from a highly mixed-race Brazilian population exhibit Class II HLA alleles associated with increased periodontal impairment. Specifically, the study identified the HLA-DRB1*03 and HLA-DRB1*15 haplogroups as being linked to severe and generalized periodontitis. These findings underscore the complex interaction between genetic factors, such as HLA polymorphisms, and systemic conditions, such as diabetes, which may act synergistically to influence the immune response and susceptibility to periodontal disease.

In addition, the findings highlight the importance of considering genetic factors, including HLA alleles, when developing personalized prevention and treatment strategies for periodontitis in genetically diverse populations such as Brazil. By identifying individuals with specific genetic profiles, clinicians can potentially tailor interventions to improve early diagnosis and management, ultimately reducing the impact of periodontitis on both oral and systemic health.

## Figures and Tables

**Table 1:** General characterization of the sample of type 1 diabetes patients included in the study.

Variables	Mean ± SD	*n* (%)
Demographic data		
Sex		
Male		23 (46.9)
Female		26 (53.1)
Age (in years)	39.7 ± 9.9	
Self-reported skin color/race		
White		8 (16.3)
Black		6 (12.2)
Mixed race		35 (71.4)
Diabetes History		
Age at diagnosis of T1D	16.5 ± 10.0	
Time since T1D diagnosis	11.2 ± 7.9	
HbA1c (%)	8.0 ± 1.6	
Fasting glucose	167 ± 103	
Periodontal Data		
Periodontitis Stage		
Stage I		16 (32.7)
Stage II		23 (46.9)
Stage III–IV		10 (20.4)
Extent of Periodontitis		
Localized (<30% of teeth)		43 (87.8)
Generalized (≥30% of teeth)		6 (12.2)
GBI (%)	4.7 ± 8.1	
VPI (%)	7.4 ± 8.8	

±SD = standard deviation. T1D = type 1 diabetes. HbA1c = glycated hemoglobin. GBI = gingival bleeding index. VPI = visible plaque index.

**Table 2:** Descriptive analysis of genetic data in the sample of type 1 diabetes patients included in the study.

Variables	Mean ± SD	*n* (%)
% Autosomal ancestry		
Native American	24.2 ± 9.4	
European	44.6 ± 15.4	
African	31.2 ± 13.5	
HLA-DRB1* haplogroup (2n)		
DRB1*01		6 (6.1)
DRB1*03		37 (37.8)
DRB1*04		22 (22.4)
DRB1*07		11 (11.2)
DRB1*08		4 (4.1)
DRB1*09		3 (3.1)
DRB1*11		4 (4.1)
DRB1*12		1 (1.0)
DRB1*13		6 (6.1)
DRB1*15		1 (1.0)
DRB1*16		3 (3.1)
HLA-DQA1* alleles (2n)		
01:01		6 (6.3)
01:02		7 (7.3)
01:03		1 (1.0)
02:01		11 (11.5)
03:01		25 (26.0)
03:02		3 (3.1)
04:01		4 (4.2)
05:01		35 (36.5)
05:05		4 (4.2)
HLA-DQB1* alleles (2n)		
02:01		33 (33.7)
02:02		15 (15.3)
03:01		5 (5.1)
03:02		24 (24.5)
03:03		1 (1.0)
03:19		1 (1.0)
04:02		5 (5.1)
05:01		7 (7.1)
05:02		1 (1.0)
06:02		1 (1.0)
06:03		1 (1.0)
06:04		3 (3.1)
06:09		1 (1.0)

±SD = standard deviation.

**Table 3:** Association of HLA-DRB1 alleles with the outcomes of extent and severity of periodontitis.

Variables	OR	(95% CI)	*p* Value
Outcome (Generalized Periodontitis)			
HLA-DRB1* haplogroup (2n)			
DRB1*01	0.23	(0.01–4.29)	0.167
DRB1*03	19.8	(1.14–346)	0.003 *
DRB1*04	0.76	(0.21–2.74)	0.680
DRB1*07	0.66	(0.13–3.26)	0.608
DRB1*08	0.36	(0.02–6.81)	0.270
DRB1*09	0.49	(0.02–9.35)	0.345
DRB1*11	2.67	(0.47–15.0)	0.251
DRB1*12	1.35	(0.06–29.8)	0.594
DRB1*13	0.23	(0.01–4.29)	0.167
DRB1*15	41.2	(1.85–917)	<0.001 *
DRB1*16	0.49	(0.02–935)	0.345
Outcome (Periodontitis Stage III–IV)			
HLA-DRB1* haplogroup (2n)			
DRB1*01	0.75	(0.15–3.76)	0.171
DRB1*03	7.71	(1.68–35.5)	0.003 *
DRB1*04	0.61	(0.21–1.77)	0.367
DRB1*07	0.32	(0.06–1.51)	0.135
DRB1*08	0.20	(0.01–3.66)	0.135
DRB1*09	0.27	(0.01–5.03)	0.200
DRB1*11	1.33	(0.24–7.17)	0.737
DRB1*12	0.74	(0.03–16.2)	0.469
DRB1*13	0.75	(0.15–3.76)	0.731
DRB1*15	21.2	(0.97–461)	0.005 *
DRB1*16	0.27	(0.01–5.03)	0.200

OR = odds ratio. 95% CI = 95% confidence interval. * *p* < 0.05 indicates statistically significant association, as determined by Chi-square or Fisher’s exact test. 2n refers to the analysis of allele frequencies based on two copies of each allele per individual.

## Data Availability

The original contributions presented in this study are included in the article, and further inquiries can be directed to the corresponding authors.

## Abbreviations

The following abbreviations are used in this manuscript:

CI	Confidence interval
CWD	Common and well-documented
GBI	Gingival bleeding index
HbA1c	glycated hemoglobin
HUUFMA	University Hospital of the Federal University of Maranhão
IL	Interleukin
OR	Odds ratio
SD	Standard deviation
T1D	Type 1 diabetes
VPI	Visible plaque index
